# Mid-term results and survival rates following a single-design rotating hinge knee arthroplasty in non-tumor conditions in a Pakistani population

**DOI:** 10.1186/s43019-021-00102-6

**Published:** 2021-05-04

**Authors:** Nouman Memon, Faizan Iqbal, Syed Shahid Noor, Kazim Rahim Najjad, Muhammad Farhan Sozera, Arsalan Abro, Noman Khan

**Affiliations:** 1 Department of Orthopaedic Surgery, Liaquat National Hospital and Medical College, National Stadium Rd, Sindh, 74800 Karachi Pakistan; 2 Department of Orthopaedic Surgery, Patel Hospital, ST-18 Block 4 Gulshan-e-Iqbal, Sindh, 75300 Karachi Pakistan

**Keywords:** Prosthetic joint infection, Instability, Arthroplasty, Rotating hinge knee, Survival

## Abstract

**Background:**

Information regarding the use of hinged implants in non-oncological conditions is limited in our region due to a lack of adequate data collection and follow-up. The purpose of this study is to evaluate mid-term results and risk factors affecting the survivorship of third-generation rotating hinge knee (RHK) patients in non-oncological conditions.

**Methods:**

We retrospectively reviewed 41 single, third-generation, rotating hinge prostheses in three complex primary knee procedures and 38 revision knee surgeries in between 2007 to 2014. Implant survival was assessed using the Kaplan-Meier method. Factors influencing implant survival were identified using the log-rank test. During the study period, clinical results along with complications were assessed. Clinical outcomes were assessed by using the Knee Society Score (KSS).

**Results:**

RHK arthroplasty was used in 41 patients. Out of 41 patients, a RHK was used in three patients with a complex primary deformed knee whereas in 38 patients, a RHK was used in revision arthroplasty surgery. The cumulative implant survival rate with re-revision due to any cause was found to be 87.8% (95% CI 69.2–90.1) at 5–7 years. Prosthetic joint infection, peri-prosthetic fracture and extensor mechanism complications were the commonest mode of failure. The *P* value was found to be significant when comparing KSS pre-operatively and post-operatively.

**Conclusion:**

The cumulative implant survival rate was found to be 87.8%. Prosthetic joint infection was the commonest mode of failure in patients who underwent third-generation RHK surgery for variable indications. Being a patient with a high Charlson comorbidity index is the main risk factor associated with failure of the rotating hinge implant.

## Background

The number of revision total knee arthroplasty (TKA) surgeries is increasing day by day due to a rise in primary TKA surgeries [1, 2]. Arthroplasty surgeons commonly face situations of bone loss and complete ligament instability during complex primary and revision surgery [3–5]. In this situation, a hinged knee implant or constrained condylar knee (CCK) could be considered. To address global instability during knee arthroplasty, the hinged implant is preferred because of concerns associated with constrained condylar knees as a CCK implant cannot overcome severe antero-posterior instability and collateral insufficiency [6, 7]. Knee stability is mainly controlled by muscles, ligaments and articular congruence. In revision surgery, there is less soft tissue support around the knee. Therefore, all stress transmitted to the implant-cement-bone interface may lead to early aseptic loosening [8]. To address this issue. a third generation of hinged implants has a rotating platform to provide some freedom at the articular surface which is important to prevent stress transmission at the implant-cement-bone interface. In revision surgery, massive bone loss indicates less surface area for contact between the residual bone and the implant. As a result, more stress transmission occurs, which ultimately leads to aseptic loosening. Traditionally, hinged implants were used in mega-prosthetic reconstructions [9, 10], but nowadays it is also reserved to address instability during complex primary and revision knee arthroplasty surgeries [6]. One previous study reports the survivorship of third-generation rotating hinge knees (RHKs) in revision knee replacement surgeries, which was found to be 50–85% at 5 years to 70–92% at 10 years [11].

There is an ongoing debate in the literature about which implant (CCK or RHK) has got better clinical outcomes and survivorship. Information regarding the use of hinged implants in non-oncological conditions is limited in our region due to a lack of adequate data collection and follow-up.

The purpose of this study was to investigate mid-term clinical outcomes and survivorship of third-generation RHK insertion after complex primary and revision TKA along with factors affecting the survival rate of hinged implants. In doing so, we also aim to determine patient-related and surgeon-related risk factors for implant failure as well as circumstances in which RHK surgery was performed in both complex primary and revision knee arthroplasty. We hypothesized that the clinical outcomes would significantly improve compared to the survival rates monitored in this study and are comparable to those of third-generation RHKs which were available in previous literature.

## Methods

This was a single-centre, retrospective study conducted at the Department of Orthopedic Surgery, Liaquat National Hospital, Karachi, Pakistan. The patients enrolled for the study were those who were operated on for complex primary and revision TKA surgery between January 2007 and December 2014. Data was principally collected from hospital records and by making telephone calls for missing information. During this period, 391 complex primary knee arthroplasties was performed in which the RHK implant was used in three cases, whereas revision knee arthroplasty surgeries were performed in 172 cases in which a RHK implant was used in 38 cases. Any primary TKA requiring more constrained implants and augments to address bone loss during the time of the index surgical procedure is considered complex primary TKA. Although this definition does not include degree of deformity, in our study we generally considered deformity to be complex primary when there is > 30° varus or valgus deformity and > 30° fixed flexion or > 10° recurvatum deformity. Forty-one patients who underwent third-generation rotating hinge implant surgery during complex primary and revision knee arthroplasty surgery to address ligament instability were identified from hospital records. The decision to use a hinged knee implant was made pre-operatively as well as during surgery. We preferred the RHK implant, especially in patients who had global instability that developed after removing the primary implant due to a septic or an aseptic cause, traumatic injury of the collateral ligaments following primary TKA or in complex deformed knees with instability in both the coronal and sagittal planes. Patients in whom a RHK implant was used in cases of primary or secondary metastatic tumor or neuromuscular disorders were excluded from the study. Patients with instability in the coronal plane only in which a CCK implant was used were also excluded from the study. Global instability is defined when there is anterior or posterior translation of the knee of > 10 mm along with medial or lateral joint opening of ≥ 10° from neutral. We used NexGen® RHKs in our study.

Factors examined include age at which surgery was performed, gender, Charlson comorbidity index (CCI), site of arthroplasty (right or left), body mass index (BMI), and primary indication for a third-generation RHK used during complex primary and revision knee arthroplasty surgery. Bone loss was classified according to the Anderson Orthopedic Research Institute System (AORI) [12]. On admission, the medical condition of all patients was assessed and classified according to the American Society of Anesthesiologists (ASA) grade [13].

The diagnosis of implant failure or aseptic loosening was made clinically and radiologically. The Modern Knee Society Radiographic Evaluation System (MKSRES) was used to diagnose loosening around the tibial and femoral components [14]. A standing scanogram for deformity assessment and a computed tomography (CT) scan to address bone loss were performed in patients who had a primary complex knee deformity as well as during revision surgery. Ultrasound color Doppler was performed for vascular assessment, especially in patients who had global instability. We used the Center for Disease Control and Prevention (CDC) criteria to diagnose prosthetic joint infection (PJI). Two-stage revision surgery was performed in all diagnosed cases of PJI.

All surgeries were performed by a single experienced arthroplasty surgeon having experience of more than 20 years. The principles of bone stock preservation, restoration of joint line, ligament balancing, and precise soft tissue dissection was followed during complex primary and revision surgery. We used the previous midline scar for exposure. The preferred approach was medial para-patellar, starting by incising the extensor retinaculum from the apex of the quadriceps tendon proximally to the tibial tubercle distally 6–8 cm from the joint line and exposing the whole of the tibial tubercle and patellar tendon. Tibial tubercle osteotomy (TTO) was performed to allow adequate exposure and implant removal in cases where the patella was unable to evert with the knee at 90° flexion. We used TTO in five cases which was then fixed with an Ethibond suture [15]. In revision cases, the hardware was removed carefully. A thorough debridement was performed, especially in infected cases which were treated with a non-articulating antibiotic cement spacer. We used 3 g vancomycin plus 3.6 g tobramycin as a cement spacer in all cases [16]. All patients remained on antibiotics for 6 weeks. All patients initially remained on intravenously administered (IV) antibiotics for 2 weeks followed by orally administered antibiotics as advised by an infectious disease (ID) consultant. Re-aspiration was considered 2 weeks after stopping antibiotics. If re-aspiration revealed no organism then revision surgery was performed after obtaining an intra-operative frozen section. Revision surgery was performed 2–3 months after first-stage revision surgery.

During revision surgery, bone loss was assessed via the AORI and managed accordingly. We used augments of variable sizes, such as 5 mm, 10 mm or 15 mm, as well as trabecular metal (TM) cones to address bone loss during the revision surgery. In type 1 AORI, where the defect is contained, we used cement to address bone loss. In type 2 AORI, if the defect is < 5 mm then we used cement or if the bone loss lay between 5 and 15 mm then we used metal augments of variable sizes. In type 3 AORI, where there is significant metaphyseal bone loss, then we used trabecular cones to address the associated bone loss. A cemented implant was used in all cases. The patella was not resurfaced in revision cases, especially if it was resurfaced previously except in cases of infection where the patella was resurfaced. In our study, the patella was resurfaced in two patients who had previously had PJI and in three patients with complex primary deformed knees. Post-operatively, a patient is encouraged to apply their full weight with crutches and to start range-of-motion exercises from the first post-operative day.

The patients were followed initially at 2 weeks, 6 weeks, 3 months, 6 months and annually thereafter till 7 years (minimum 5 years and maximum 7 years). Functional outcome was measured annually using the Knee Society Score (KSS) at 1 year and at final follow-up [17]. At each follow-up visit, the patients were asked about possible complications and were evaluated clinically as well as radiologically. Patients’ radiographs were compared with the earliest radiograph performed post-operatively to look for signs of loosening [14].

Poly of the hinge knee with rotating platform has a stem that actually resides within the tibial tray and is free to rotate. This helps to take most of the stress away from the implant-cement-bone interface, thereby preventing the risk of loosening. Keeping this concept in mind, our study has two end points.

The first end point is to evaluate the survival of third-generation RHKs by observing the time of index surgery to re-operation due to failure from any cause such as aseptic loosening, PJI, extensor mechanism complications and peri-prosthetic fracture. The second end point is to determine the risk factors that mainly influence the survival of a RHK. Both patient-related and surgeon-related factors were evaluated in order to find out the independent factor that mainly influences implant survival. Age, comorbidities, BMI, ASA score, CCI and indications for RHK insertion were the main risk factors that were evaluated.

### Statistical analysis

A power analysis was performed to estimate sample size. By using PASS11 for the clinical score, we found that a sample size of 41 achieves 100% power to detect a mean of paired differences of − 27.9 with an estimated standard deviation of differences of 2.7 and with a significance level (alpha) of 0.05000 using a two-sided paired *t* test. By using PASS11 for the functional score, we found that a sample size of 41 achieves 100% power to detect a mean of paired differences of − 32.0 with an estimated standard deviation of differences of 0.4 and with a significance level (alpha) of 0.05000 using a two-sided paired *t* test. Implant survival analysis was performed using the Kaplan-Meier method [18]. Factors influencing implant survival were assessed using the log-rank test [19] and 95% confidence intervals were calculated. Continuous variables were analyzed using Fischer’s exact test. A *P* value < 0.05 was considered significant.

## Results

### Demographic characteristics

A RHK was used in 41 patients. Three patients with complex primary deformed knees underwent RHK surgery, whereas 38 patients underwent RHK insertion for revision surgery. Out of 41 patients, 12 (29.2%) patients were male whereas 29 (70.7%) patients were female. Right knee arthroplasty was performed in 18 (43.9%) patients, whereas left knee arthroplasty was performed in 22 (53.6%) patients and bilateral arthroplasty surgery was performed in only 1 (2.43%) patient. The mean BMI was 29.2 kg/m^2^. Out of 41 patients, 11 (26.8%) patients were diabetic, 15 (36.5%) were hypertensive, 3 (7.31%) patients had ischemic heart disease (IHD), 4 (9.7%) patients had rheumatoid arthritis (RA), 3 (7.31%) patients had asthma, 4 (9.7%) patients had more than two comorbidities, whereas only 1 (2.43%) patient had no comorbidity. The common indication for a RHK was aseptic loosening of the primary knee implant in 25 (60.9%) cases followed by global instability in 11 (26.8%) cases, PJI in 2 (4.87%) cases, and complex deformed knees in 3 (7.31%) cases. Twenty-nine (70.7%) patients had an ASA score of 3 whereas 12 (29.2%) patients had an ASA score of 2 in our study. We commonly encountered tibial-side bone loss with AORI class 2a in 27 (65.8%) patients. The detailed demographic results are presented in Table 1.

**Table 1:** Descriptive statistics of demographics

Variables	Number (***n*** = 41)
Age (years)	66.4 (range 51–74)
Sex	
Male	12 (29.2%)
Female	29 (70.7%)
Site of arthroplasty	
Right	18 (43.9%)
Left	22 (53.6%)
Bilateral	1 (2.43%)
Body mass index (BMI)	29.2 (range 20.2–50.2)
Comorbidities	
Diabetes mellitus (DM)	11 (26.8%)
Hypertension (HTN)	15 (36.5%)
Ischemic heart disease (IHD)	3 (7.31%)
Rheumatoid arthritis (RA)	4 (9.7%)
Asthma	3 (7.31%)
SLE	–
> 2 comorbidities	4 (9.7%)
No comorbidities	1 (2.43%)
Indications for the rotating hinge knee (RHK)	
**A. Revision total knee arthroplasty**	
Aseptic loosening of primary knee implant	25 (60.9%)
Prosthetic joint infection after primary TKA	2 (4.87%)
Global instability following primary TKA	11 (26.8%)
**B. Complex primary knees with bone loss**	
Varus deformity (> 30°)	1 (2.43%)
Valgus deformity (> 30°)	1 (2.43%)
Fixed flexion deformity (> 30°)	1 (2.43%)
Recurvatum deformity (> 10°)	–
Deformity in > 1 plane	**–**
AORI classification for bone loss	
T1	4 (9.75%)
T2a	27 (65.8%)
T2b	1 (2.43%)
T3	2 (4.87%)
F1	3 (7.31%)
F2a	2 (4.87%)
F2b	1 (2.43%)
F3	1 (2.43%)

*AORI* Anderson Orthopedic Research Institute Classification System, *T* tibia, *F* femur, *SLE* systemic lupus erythematosus, *TKA* total knee arthroplasty

### Functional outcome

The clinical score was found to be 52.21 ± 4.05, whereas it was 79.42 ± 2.2 and 80.12 ± 1.33 post-operatively after 1 year and at the final follow-up. The functional score was 49.33 ± 3.24 pre-operatively, whereas it was 80.28 ± 2.99 and 81.34 ± 2.82 post-operatively at the first year and at the final follow-up. The *P* value was found to be significant when comparing the KSS pre-operatively and post-operatively as shown in Table 2.

**Table 2:** Knee Society score (KSS) clinical and functional scores

Parameters	Pre-operative	Post-operative at 1 year	Post-operative at final follow-up	***P*** value
Clinical scores (out of 100)	52.21 ± 4.05	79.42 ± 2.2	80.12 ± 1.33	**0.002**
Functional scores (out of 100)	49.33 ± 3.24	80.28 ± 2.99	81.34 ± 2.82	**0.001**

*P* value **<** 0.05 considered significant

### Implant survival

**Fig. 1:**
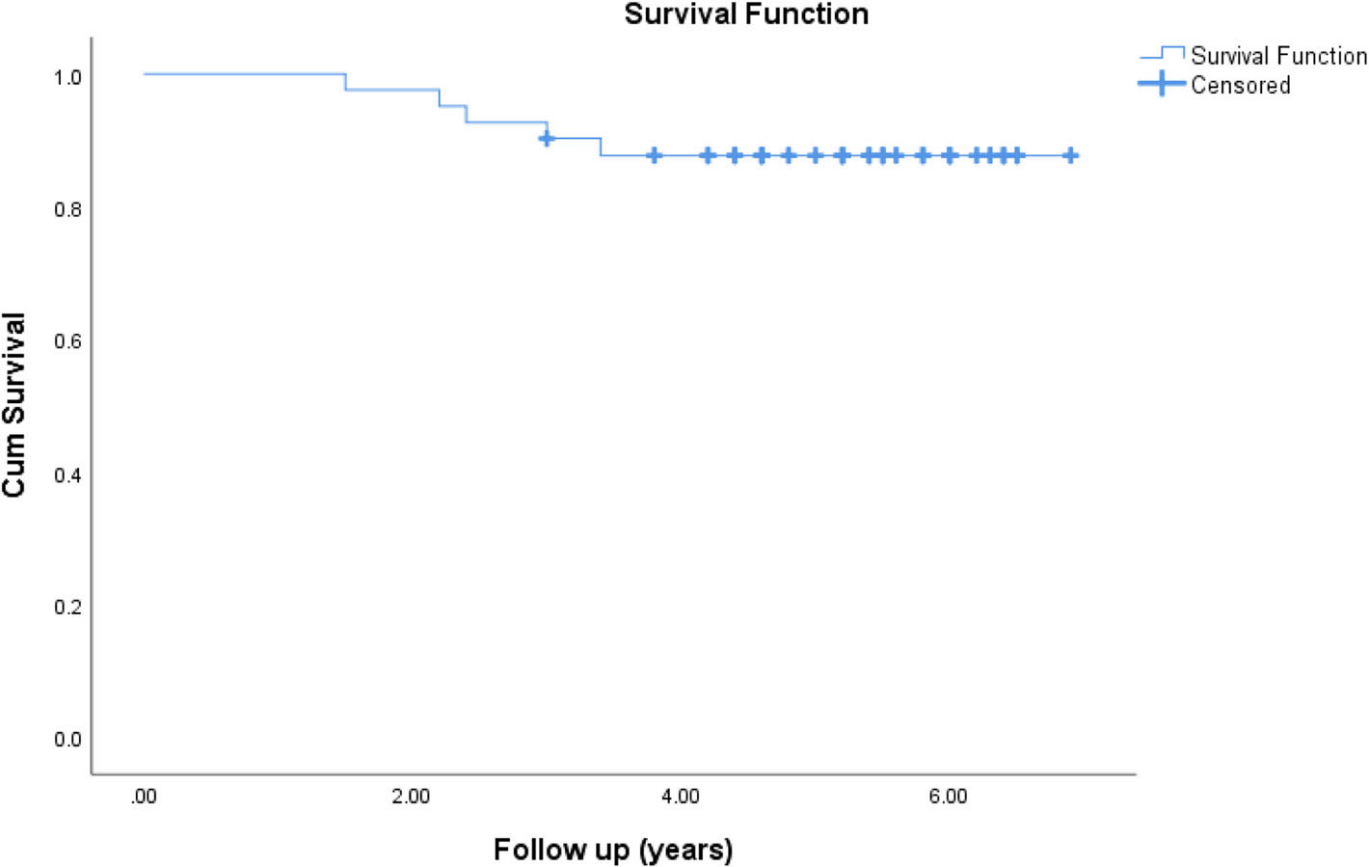
Cumulative survival of the rotating hinge knee using Kaplan-Meier survival analysis

Implant survival was measured by Kaplan-Meier survival analysis. The survival analysis is plotted in Fig. 1. The x-axis shows the duration of follow-up whereas the y-axis shows the cumulative survival of the implants. Data of patients who did not experience any event during the study period or were lost to follow-up before experiencing an event was kept censored. In our study, only five patients had an event at a different interval during follow-up. The first implant failure occurred after 20 months as shown by the drop in the height of the survival function curve. The survival rate remains static after 4 years from the time of the index surgery. The cumulative implant survival rate with re-revision due to any cause was found to be 87.8% (95% CI 69.2–90.1) at 5–7 years. The reasons for implant failure were non-mechanical, such as PJI in three patients, followed by peri-prosthetic fracture and extensor mechanism complications in one patient each. Two patients were lost to follow-up before experiencing an event and were kept as censored. No deaths occurred during the study period.

Multiple factors were retrieved from the data in order to identify any independent risk factor that mainly affects the survival of the implant. Age, comorbidities, site of arthroplasty, BMI, ASA score and indications of surgery had no impact on the survivorship of the implants. Patients with a high CCI had an impact on implant survivorship as shown in Table 3.

**Table 3:** Factors affecting implant survival

Variable	Patients (***n*** = 41)	5-year implant survival in %	95% confidence interval	***P*** (log-rank)
**Age at surgery**				
> 60	22	74%	69–80	n/s
< 60	19	79%	73–86	n/s
**Comorbidities**				
Diabetes mellitus (DM)	11 (26.8%)	77%	70–84	n/s
Hypertension (HTN)	15 (36.5%)	82%	71–87	n/s
Ischemic heart disease (IHD)	03 (7.31%)	80%	69–83	n/s
Rheumatoid arthritis (RA)	04 (9.7%)	70%	69–81	n/s
Asthma	03 (7.31%)	78%	74–81	n/s
SLE	0	84%	78–87	n/s
> 2 comorbidities	04 (9.7%)	71%	69–85	n/s
No comorbidities	01 (2.43%)	70%	69–81	n/s
**Charlson comorbidity index (CCI) score**				
**0–2**	**1 (2.4%)**	89%	84–89	n/s
**3–5**	**5 (12.1%)**	82%	79–85	n/s
**6–8**	**35 (85.3%)**	53%	49–55	Significant
**Site of arthroplasty**				
Right	18 (43.9%)	71%	69–76	n/s
Left	22 (53.6%)	77%	73–84	n/s
Bilateral	01 (2.43%)	81%	77–82	n/s
**Body mass index** (BMI)				
> 30	17 (41.4%)	79%	77–82	n/s
< 30	24 (58.5%)	84%	79–86	n/s
**ASA score**				
Class 2	12 (29.2%)	81%	78–84	n/s
Class 3	29 (70.7%)	85%	81–87	n/s
**Indications for rotating hinge knee (RHK) surgery**				
Global instability	11 (26.8%)	87%	82–88	n/s
Aseptic loosening	25 (60.9%)	70%	69–73	n/s
PJI	02 (4.87%)	82%	79–81	n/s
Complex primary	03 (7.31%)	84%	80–88	n/s

*ASA* American Society of Anesthesiologists, *n/s* not significant, *PJI* prosthetic joint infection

## Discussion

The purpose of our study was to evaluate mid-term survivorship of third-generation hinge knees with a rotating platform in non-oncological conditions, especially in developing countries like Pakistan. Previously, hinged implants were mainly used as a salvage procedure, especially in low-demand patients with severe knee instability. This is because of an increased rate of failure due to mechanical complications, such as aseptic loosening, but with the innovation of third-generation rotating hinge implants with a rotating platform, there is less stress transmission at the bone-cement-implant interface which may improve the overall survivorship of rotating hinge implants. To the best of our knowledge, this study is first from a developing country like Pakistan to report the results of mid-term survivorship of third-generation RHKs. There is an ongoing debate regarding the survivorship and clinical outcomes of CCK and RHK surgery. Previous meta-analysis reveals that RHKs have higher short-term (< 5 year) survival rates and lower mid-term (5–10 years) survival rates than CCK surgery. The difference in terms of survivorship is not statistically significant. In contrast to CCK, which acts as a constrained implant in the coronal plane only, the rotating hinge knee allows more freedom in the axial plane and also acts as a more constrained implant in both the coronal and sagittal planes [20]. The cumulative survival of third-generation RHKs in the current study was 87.8% (95% CI 69.2–90.1) at 5–7 years. The main reason for implant removal in our study was non-mechanical, such as peri-prosthetic joint infection in 3 (60%) cases followed by peri-prosthetic fracture and extensor mechanism complication in 1 (20%) case each. We did not encounter any mechanical complication, such as aseptic loosening, in our patients till 5 years from the index surgery which was one of the most important findings of our study. Theil et al. [21] also report the cumulative survival of third-generation RHKs in complex primary as well as in revision knee surgeries due to failure of primary knee arthroplasty. The overall cumulative survival of third-generation RHKs with implant removal due to any cause was found to be 91.3% (95% CI 86.4–96.2) after 2 years and 69.7% (95% CI 60.9–78.5) after 5 years. They also report that PJI was found to be the major cause of implant removal in 20 (15%) of cases followed by aseptic loosening in 12 (9%) cases and peri-prosthetic fracture in 2 (2%) cases [21].

We did not encounter any needs for re-revision due to any cause in the first year from the index surgery and no event occurred after 4 years. This shows that implant removal due to failure by any cause was needed between 20 months and 4 years in this study. We commonly found PJI to be one of the most important causes of implant removal during our study period which was actually not surprising for us as all the patients had systemic disease in our study except for one, which might be the reason for failure due to infection in the revision surgery. We did not encounter any complications in complex primary cases. All the complications were encountered in revision surgery. Previous studies also report PJI to be the most common mode of failure after RHK surgery followed by aseptic loosening after 10 years [11, 22].

We also found that patients who underwent RHK implantion during revision surgery who had a high CCI are more prone to have failed outcomes. This might be due to the thin soft tissue envelope that forms at the surgical site due to the multiple previous surgeries. These findings are similar to the findings observed in a previous study which also confirmed that patients with a high CCI are at increased risk for failure following RHK arthroplasty [21, 22].

Smith et al. [11] performed a comparative analysis between mechanical and non-mechanical complications as a cause of failure, especially in patients who underwent third-generation RHK surgery in non-oncological conditions. They concluded that the overall survivorship of RHKs was 54% at 4 years with non-mechanical causes being the most common mode of failure. Although our results were superior in terms of survivorship, non-mechanical failure was also the most common mode of failure in our study. Springer et al. [23] also found non-mechanical failure to be the most common mode of failure in 26 (19.2%) cases.

Implant fixation is generally dependent on the use of cemented as well as uncemented stems. We used cemented implants in all our patients who underwent third-generation hinged implant surgery with a rotating platform regardless of age. The mean age in our study was 66.4 years (range 51–74 years). Fleischman [24] performed a comparative analysis between cementless and cemented stems in patients who underwent revision surgery. They found that the risk of mechanical failure was equivalent in both groups. Although there was an increasing trend for failure or mechanical loosening with cemented stems in patients aged < 65 years, statistically it was not significant. Farid et al. [25] used a cemented implant only in cases with poor bone quality and found an increased rate of loosening. Based on previous studies, there is still no agreement as to which mode of fixation should be used in revision surgeries. We believe that a more comprehensive comparison must be performed in order to make a final consensus regarding mode of fixation.

In our study, functional outcome was assessed using the KSS. Pre-operatively, the clinical score was found to be 52.21 ± 4.05 and the functional score was 49.33 ± 3.24. There was a marked improvement in clinical and functional scores post-operatively at the final follow-up with a *P* value < 0.05 as shown in Table 2. Kearns et al. [26] in his study reported the results of third-generation hinged implants with a rotating platform in complex primary and revision knee surgeries. The mean KSS improved from 35.7 to 66.2 points at 5 years. Implant survival was found to be 70.7% at 5 years. Previously, a prospective analysis was performed in order to evaluate clinical and radiological outcomes of RHK implants in patients with gross ligamentous instability. They found a significant improvement in the clinical and functional scores at final follow-up [27].

There are essentially three generations of hinged devices. The first generation of hinged designs allows motion in only one plane, such as flexion and extension, but there is no rotation that results in stress transmission to the bone-implant interface causing early failure of the implant due to aseptic loosening. This prompted the development of the second generation hinged design to provide rotation in order to prevent stress transmission at the bone-implant interface. Second generation implants show good to excellent clinical results but show unacceptable complication rates of up to 80% in the previous literature. Both first-generation and second-generation hinged designs were then replaced with the third-generation rotating hinge design [28].

There were several limitations to our study. The sample size was limited to 41 patients which means that only a small number of surgeries were performed during the study period. We mainly focused on one type of hinged implant in our study, which is in contrast with other studies that focused on different types of hinged implant designs. The current study is not a comparative study between two implants such as the RHK and the CCK. Future studies are required to investigate whether RHK or CCK prostheses lead to better clinical outcomes and survival rates. Furthermore, this study was retrospective and a 10-year follow-up must be required in order to evaluate the survivorship of rotating hinge knee implants.

## Conclusion

Our study concludes that survivorship of third-generation RHKs with a rotating platform in non-oncological conditions was found to be 87.8%. Patients with a high CMI are the main risk factor associated with failure of the RHK. Non-mechanical complications, such as PJI, were the major mode of failure in patients who underwent RHK design procedures for variable indications.

## Data Availability

Not applicable

## Abbreviations

RHK: Rotating hinge knee
KSS: Knee Society Score
BMI: Body mass index
AORI: Anderson Orthopedic Research Institute System
ASA: American Society of Anesthesiologists
CCI: Charlson comorbidity index
MKSRES: Modern Knee Society Radiographic Evaluation System
CT scan: Computed tomography
CDC: Center for Disease Control and Prevention
PJI: Prosthetic joint infection
TTO: Tibial tubercle osteotomy
TM: Trabecular metal
IHD: Ischemic heart disease
RA: Rheumatoid arthritis
